# Screening topological materials with a CsCl-type structure in crystallographic databases

**DOI:** 10.1107/S2052252519007383

**Published:** 2019-06-13

**Authors:** L. Jin, X. M. Zhang, X. F. Dai, L. Y. Wang, H. Y. Liu, G. D. Liu

**Affiliations:** a School of Materials Science and Engineering, Hebei University of Technology, Tianjin 300130, People’s Republic of China; b Tianjin Key Laboratory of Low Dimensional Materials Physics and Preparation Technology, School of science, Tianjin University, Tianjin 300354, People’s Republic of China

**Keywords:** CsCl-type materials, crystal structures, electronic structures, first-principles calculations, inorganic materials, density functional theory, topological modeling

## Abstract

CsCl-type materials have many outstanding characteristics, *i.e.* simple in structure, ease of synthesis and good stability at room temperature, thus are an excellent choice for designing functional materials. Using high-throughput first-principles calculations, a large number of topological semimetals/metals (TMs) were designed from CsCl-type materials found in crystallographic databases and their crystal and electronic structures have been studied. The CsCl-type TMs in this work show rich topological character, ranging from triple nodal points, type-I nodal lines and critical-type nodal lines, to hybrid nodal lines. The TMs identified show clean topological band structures near the Fermi level, which are suitable for experimental investigations and future applications. This work provides a rich data set of TMs with a CsCl-type structure.

## Abstract 2

A large number of topological materials with a CsCl-type structure in crystallographic databases are explored and their crystal and electronic structures are studied.

## 1. Introduction   

Band topology in insulating and semi-metallic/metallic materials has received intense research enthusiasm in recent years. Such enthusiasm was initially generated by the discovery of topological insulators (Hasan & Kane, 2010 ▸; Qi & Zhang, 2011 ▸), which show novel gapless surface states associated with nontrivial band topology. Recently, motivated by the notable progress on Weyl semimetals (Wan *et al.*, 2011 ▸; Murakami, 2007 ▸; Burkov & Balents, 2011 ▸; Weng, Fang *et al.*, 2015 ▸) and Dirac semimetals (Wang *et al.*, 2012 ▸, 2013 ▸; Young *et al.*, 2012 ▸; Yang & Nagaosa, 2014 ▸), special attention has been attracted onto topological semimetals/metals (TMs). TMs feature with nontrivial band crossing near the Fermi level. Their band crossing can show different configurations such as Weyl type (Wan *et al.*, 2011 ▸; Murakami, 2007 ▸; Burkov & Balents, 2011 ▸; Weng, Fang *et al.*, 2015 ▸), Dirac type (Wang *et al.*, 2012 ▸, 2013 ▸; Young *et al.*, 2012 ▸; Yang & Nagaosa, 2014 ▸), multiple-nodal-point type (Soluyanov *et al.*, 2015 ▸; Bradlyn *et al.*, 2016 ▸; Zhu *et al.*, 2016 ▸), nodal-line type and so on (Weng, Liang *et al.*, 2015 ▸; Chen *et al.*, 2015 ▸; Wu, Liu *et al.*, 2018 ▸; Zhang, Yu, Zhu *et al.*, 2018 ▸; Zhang, Guo *et al.*, 2017 ▸; Yang *et al.*, 2014 ▸), giving rise to rich TM phases. TMs have shown remarkable properties ranging from protected surface states, rich magnetotransport properties (Fang *et al.*, 2003 ▸; Weng, Yu *et al.*, 2015 ▸; Singha *et al.*, 2017 ▸; Ali *et al.*, 2016 ▸) and unusual optical responses (Yu *et al.*, 2016 ▸; Guan *et al.*, 2017 ▸; Liu *et al.*, 2017 ▸), to high-temperature superconductivity (Zhang *et al.*, 2016 ▸; Chang *et al.*, 2016 ▸; Pang *et al.*, 2016 ▸; Guan *et al.*, 2016 ▸).

Intense effort has been made in exploring TMs in realistic materials. Among various TMs, Weyl and Dirac semimetals have been well studied both theoretically and experimentally (Wan *et al.*, 2011 ▸; Murakami, 2007 ▸; Burkov & Balents, 2011 ▸; Weng, Fang *et al.*, 2015 ▸; Wang *et al.*, 2012 ▸, 2013 ▸; Young *et al.*, 2012 ▸; Yang & Nagaosa, 2014 ▸; Fang *et al.*, 2003 ▸; Weng, Yu *et al.*, 2015 ▸). For other types of TMs, especially nodal-line and triple-nodal-point TMs, although numerous candidate materials are theoretically predicted, their experimental progress is still far from satisfaction. Among the proposed nodal-line TMs (Kim *et al.*, 2015 ▸; Yu *et al.*, 2015 ▸; Yamakage *et al.*, 2016 ▸; Huang *et al.*, 2016 ▸; Schoop *et al.*, 2016 ▸; Neupane *et al.*, 2016 ▸; Hu *et al.*, 2016 ▸; Du *et al.*, 2017 ▸; Li *et al.*, 2016 ▸; Hirayama *et al.*, 2016 ▸; Zhang, Jin, Dai & Liu, 2018 ▸; Zhang, Jin *et al.*, 2017 ▸; Xu *et al.*, 2017 ▸; Zhang, Yu *et al.*, 2017 ▸; Feng *et al.*, 2018 ▸; Zhang, Yu, Lu *et al.*, 2018 ▸; Zhang, Jin, Dai *et al.*, 2018 ▸; Sheng & Nikolić, 2017 ▸; Liu, Jin *et al.*, 2018 ▸; Zhu *et al.*, 2018 ▸; Zhang, Yu *et al.*, 2017 ▸; Li *et al.*, 2017 ▸), the nodal-line signature has only been detected in a few materials including ZrSiS (Schoop *et al.*, 2016 ▸; Neupane *et al.*, 2016 ▸; Hu *et al.*, 2016 ▸), PbTaSe_2_ (Yu *et al.*, 2018 ▸; Bian *et al.*, 2016 ▸) and *M*B_2_ (*M* = Ti, Zr and Al) (Liu, Lou *et al.*, 2018 ▸; Takane *et al.*, 2018 ▸; Yi *et al.*, 2018 ▸). For triple-nodal-point TMs, only the compound MoP has been confirmed by experiments (Lv *et al.*, 2017 ▸). This is because ideal nodal line TMs and triple nodal point TMs that are suitable for experimental investigation are scarce. To favor experimental detection, from a materials point of view, at least the following two conditions need to be satisfied: first, the material possesses a clean band structure so that its band crossing does not coexist with other interfering bands; second, the material should be easy to synthesize and have good stability. Currently, there is an urgent need to exploit excellent nodal-line TMs and triple-nodal-point TMs suitable for experimental detection.

**Figure 1:**
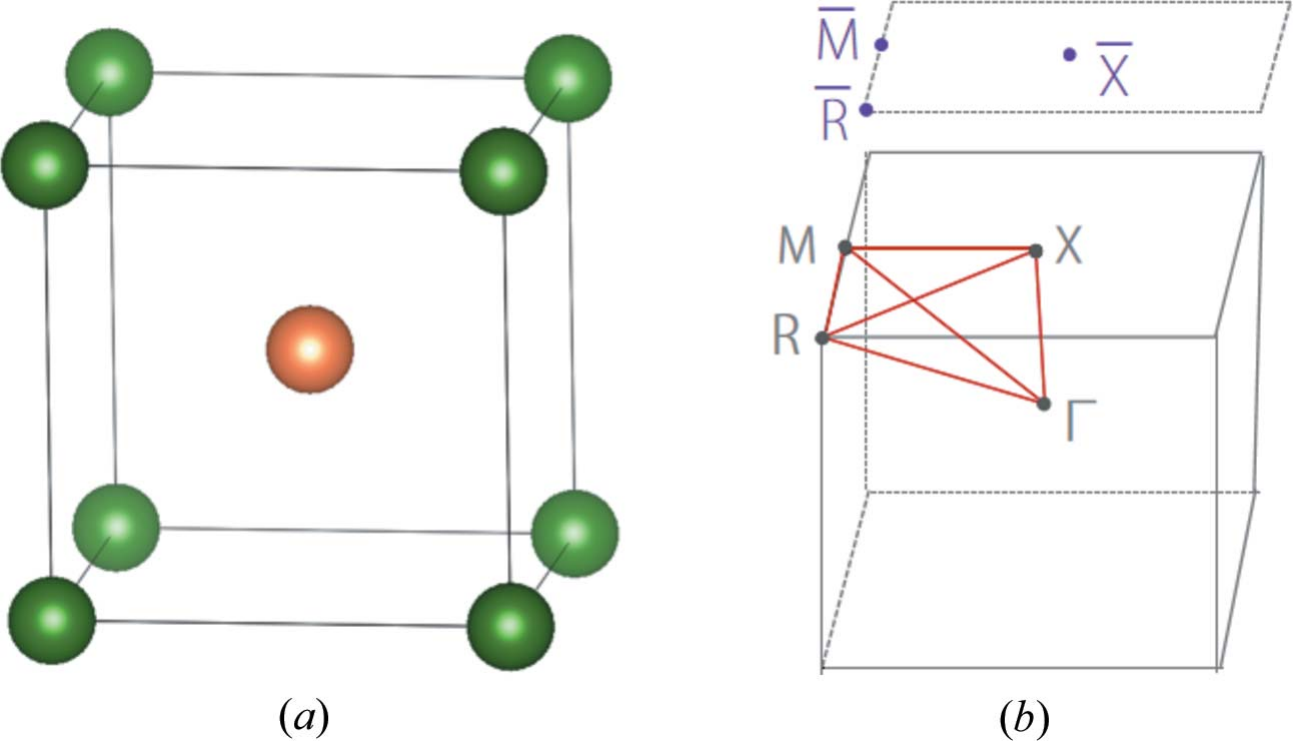
(*a*) CsCl-type crystal structure and (*b*) its Brillouin zone of bulk and the (001) surface.

In this work, by performing high-throughput first-principles calculations, we systematically screen TMs from existing CsCl-type materials. As shown in Fig. 1 ▸(*a*), CsCl-type materials have a simple crystal structure that possesses only two atomic positions, known as *1a* (0, 0, 0) and *1b* (1/2, 1/2, 1/2) Wyckoff sites. CsCl-type materials are quite easy to synthesize, and most of them can be directly prepared from melting without any further chemical treatment (Zimmer *et al.*, 1985 ▸; Eremenko *et al.*, 1966 ▸; Blazina *et al.*, 1989 ▸; Smith *et al.*, 1965 ▸). Moreover, CsCl-type materials usually have good stability, and it is found that there exists more than 140 CsCl-type materials stable at room temperature. Therefore, CsCl-type materials are good candidates for exploring TMs. Our material screening has identified 61 TMs from CsCl-type materials, among which, 15 triple-nodal-point TMs, 39 nodal-line TMs, 7 TMs coexisting as nodal-line and triple-nodal-point have been obtained. In particular, the identified nodal-line TMs exhibit various types of band dispersion slope, ranging from type-I, critical-type to type-II and a hybrid type. Their electronic band structures and nontrivial surface states are systemically studied. Our results provide a rich data set of triple-nodal-point and nodal-line TMs suitable for experimental study.

## 2. Methods   

**Figure 2:**
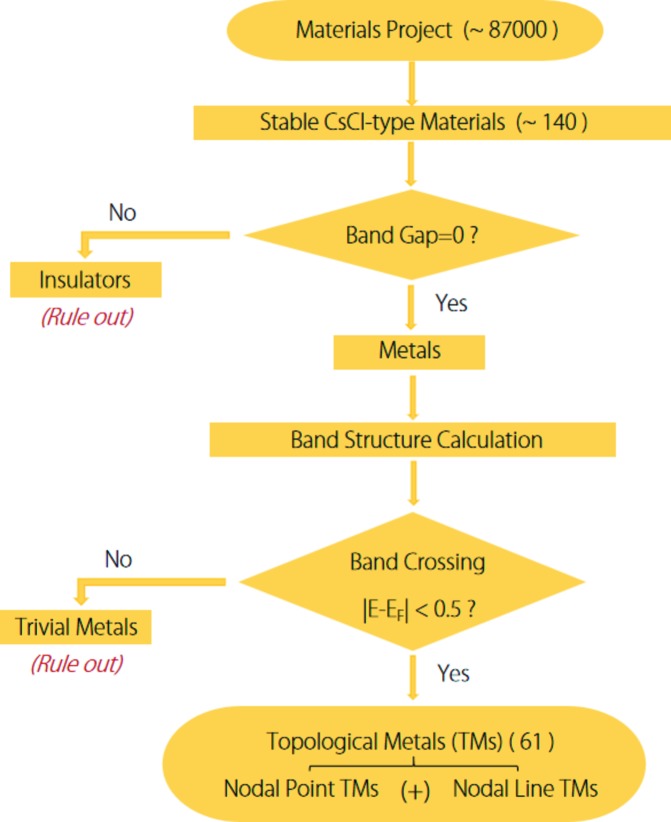
Flowchart for screening topological metals in CsCl-type materials.

Materials screening is performed using high-throughput first-principles calculations, and the corresponding screening process is shown in Fig. 2 ▸. We first screen out stable CsCl-type materials from the Materials Project data (with ∼87 000 entries) (Jain *et al.*, 2013 ▸); then we exclude the insulating CsCl-type materials by evaluating the band gap, and those with metallic band structures (no band gap) are selected for further computation. We perform band structure calculations to detect possible band crossing, which is essential for forming TMs. Here only the band crossing at |*E* − *E*_F_| < 0.5 eV is considered because the topological signature in this region is the most promising to be detected in experiments. As a result, we obtained 61 CsCl-type TMs with triple-nodal-point or/and nodal-line character.

Our calculations were carried out using the Vienna *ab initio* Simulation Package (*VASP*; Kresse & Joubert, 1999 ▸; Kresse & Hafner, 1993 ▸). For the exchange-correlation potential, we used the generalized gradient approximation (GGA) of the Perdew–Burke–Ernzerhof formalism (Perdew *et al.*, 1996 ▸). The cutoff energy was chosen to be 500 eV, and the Brillouin zone (BZ) was sampled with a Γ-centered *k*-mesh of 13 × 13 × 13. For materials containing transition-metal and rare-earth elements, we executed GGA + *U* calculations to describe the Coulomb interaction (Anisimov *et al.*, 1991 ▸; Dudarev *et al.*, 1998 ▸). The effective Coulomb energy *U*_eff_ was set at 2.5 eV for Sc and Ti, 3 eV for Y, Cd, Pd, Os, Ir and Hf, and 5 eV for Tm, Ho, Dy, Tb, Pr and Yb. The results do not change with slight changes in the value of *U*_eff_. The topological surface states are calculated based on the maximum-localized Wannier functions (Marzari & Vanderbilt, 1997 ▸; Mostofi *et al.*, 2008 ▸) using the *WANNIERTOOLS* package (Wu, Zhang *et al.*, 2018 ▸).

## 3. Results and discussion   

### 3.1. Screened TMs   

**Figure 3:**
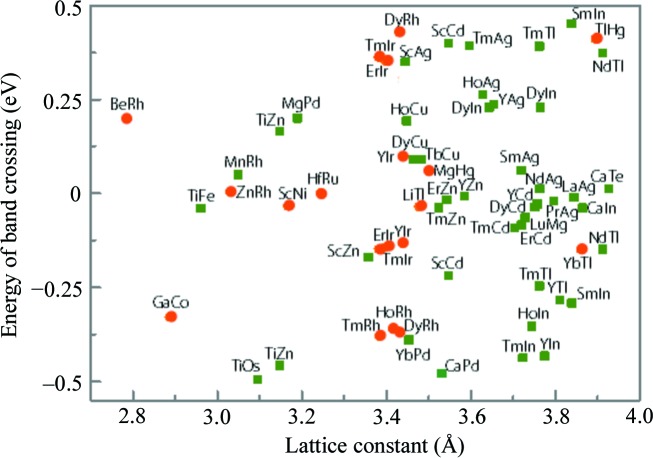
Identified TMs with triple-nodal-point (orange circles) and nodal-line (green squares) in CsCl-type materials. The longitudinal ordinate is the energy position of band crossing for the triple nodal point and nodal line. The horizontal ordinate is the equilibrium lattice constant.

**Figure 4:**
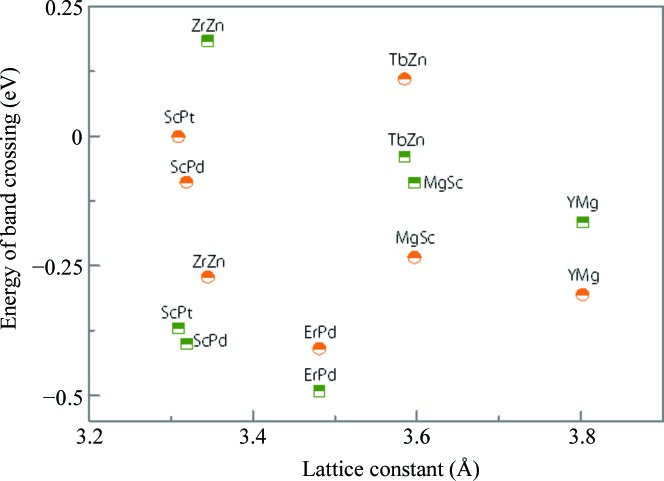
Identified TMs with coexisting nodal line and triple nodal point. The energy positions of band crossing for the triple nodal point and nodal line are shown with orange circles and green squares, respectively.

Our material screening has identified 61 TMs in CsCl-type materials. Among them, 54 TMs exhibit triple-nodal-point or nodal-line band crossing. Their material composition, lattice constants and the energy positions of band crossings are summarized in Fig. 3 ▸. In addition, we found that 7 CsCl-type materials (ScPt, ScPd, ZrZn, ErPd, TbZn, MgSc and YMg) possess triple-nodal-point and nodal-line characters simultaneously, and the results are shown in Fig. 4 ▸. Using Figs. 3 ▸ and 4 ▸, one can conveniently recognize the topological signature of the identified CsCl-type TMs.

In the following, for the identified TMs with different topological characters, we provide typical examples for detailed discussion, where YIr represents triple-nodal-point TMs, CaTe and TiOs are type-I nodal-line TMs, CaPd are critical-type nodal-line TMs, YCd are hybrid nodal-line TMs and YMg are TMs of coexisting nodal-line and triple-nodal-point signatures.

### 3.2. Triple-nodal-point TMs   

**Figure 5:**
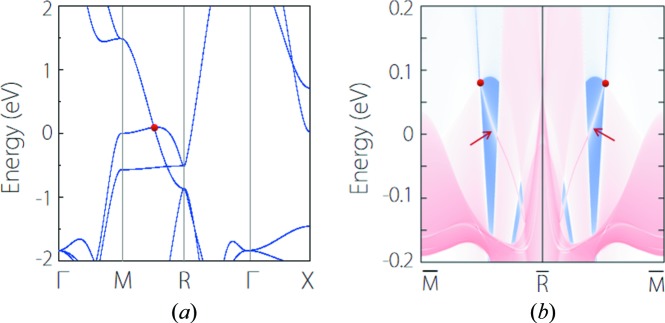
(*a*) Electronic band structure of YIr. The red dot denotes the triple nodal point. (*b*) Projected spectrum on the (001) surface of YIr. The red arrows point to the Fermi arc originating from the triple nodal point.

YIr is a typical triple-nodal-point TM; its electronic band structure is shown in Fig. 5 ▸(*a*), which clearly shows a band-crossing point at the *M–R* path near the Fermi level (at *E* − *E*_F_ = 0.08 eV). Excluding spin, this band-crossing point has a triple degeneracy formed by one non-degenerate band and one doubly degenerate band. It should be noted that, with the exception of the bands which form the triple nodal point, there exists no other extraneous band nearby. Such a clean band structure generally favors experimental detection of triple nodal points in YIr. One of the representative signatures of triple-nodal-point TMs is the existence of Fermi-arc surface states (Zhu *et al.*, 2016 ▸; Yang *et al.*, 2017 ▸; Weng *et al.*, 2016 ▸; Jin *et al.*, 2019 ▸). For YIr, we show the (001) surface spectrum in Fig.5(*b*). We can clearly observe the Fermi-arc states originating from the triple nodal points. It is worth noting that, since the triple nodal points are nearly situated in the middle of the *M–R* path, the Fermi arc in YIr spans a large scale of momenta. Evidence of Fermi-arc states in YIr can be readily detected by experiment.

### 3.3. Nodal-line TMs   

**Figure 6:**
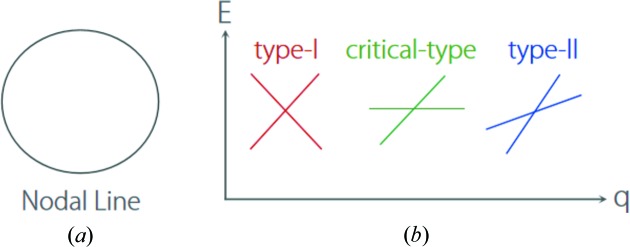
Schematic illustrations of (*a*) a nodal line and (*b*) type-I, critical-type and type-II band dispersions in the momentum–energy space.

For nodal-line TMs, as shown in Fig. 6 ▸(*a*), the band crossing forms a one-dimensional nodal line. On the nodal line, each point exhibits linear band crossing. According to the slope of band dispersion, band crossing can be termed as three types, namely type-I, critical-type and type-II, as shown in Fig. 6 ▸(*b*). For type-I, the bands have conventional dispersion, where the electron-like and hole-like states are completely separated by the crossing point. For type-II, the conical spectrum is tipped over. As a result, the electron-like and hole-like states coexist at each energy level. For critical type, which is formed by a flat band and a dispersive band (Liu, Jin *et al.*, 2018 ▸), it has the critical band dispersion between conventional type-I and type-II. Based on these types of band crossing, nodal line can also be termed as type-I, critical-type and type-II, where each point on the nodal line has type-I, critical-type and type-II band crossing, respectively. The nodal line can also have a fourth possibility where type-I and type-II band crossings coexist in one nodal line. Such nodal-line state usually occurs when one of the crossing bands possesses a saddle-like dispersion, and was termed a hybrid nodal line (Zhang, Yu, Lu *et al.*, 2018 ▸; Gao *et al.*, 2019 ▸).

**Figure 7:**
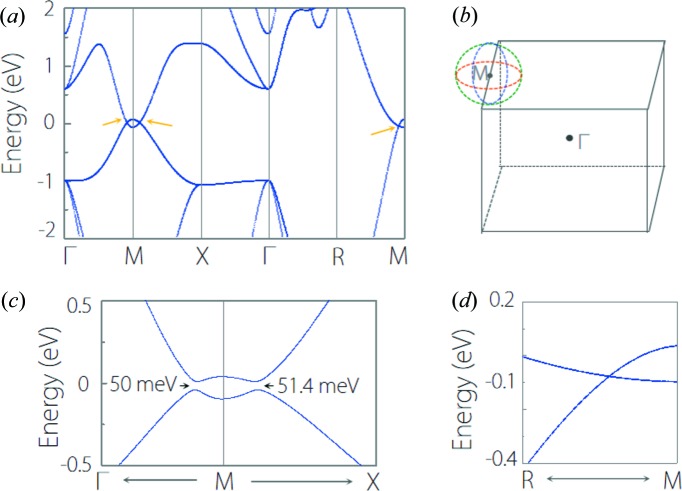
(*a*) Electronic band structure of CaTe without SOC. (*b*) Schematic illustration of the three crossing nodal lines in CaTe. (*c*) and (*d*) show the enlarged band structure of CaTe with SOC at the Γ–*M*–*X* and *R*–*M* paths, respectively.

We found that CsCl-type materials show rich nodal-line band structures. Among them, CaTe is a typical type-I nodal-line TM. The band structure of CaTe without spin-orbit coupling (SOC) is shown in Fig. 7 ▸(*a*). At the Γ–*M*, *M*–*X* and *R*–*M* paths, there exists type-I band-crossing points, as indicated by the arrows in Fig. 7 ▸(*a*). Because of the protection of the inversion symmetry (*P*) and the time reversal symmetry (*T*) in the CaTe system, these crossing points in fact reside on nodal lines centering on the *M* point. By performing more detailed computations, we find the nodal line is situated in the *k*_z_ = π plane, under additional protection of the mirror symmetry (*M*_z_). Considering the cubic symmetry of the CaTe lattice, the system possesses in total three equivalent nodal lines crossing each other, as shown in Fig. 7 ▸(*b*). CaTe is an excellent type-I nodal-line TM because of the following: (1) it has a clean nodal-line band structure; (2) the nodal lines are quite close to the Fermi level; and (3) the crossing bands have a very large linear energy range (∼2 eV). With the SOC effect, the nodal line is gapped beside a pair of Dirac points leaving at the *R–M* path (protected by the *C*_4_ rotation symmetry), as shown in Figs. 7 ▸(*c*) and 7 ▸(*d*). In fact, Du *et al.* (2017 ▸) reported that CaTe with a CsCl-type structure is a node-line semimetal when the SOC is neglected. They found that three node-line rings are perpendicular to one another around the *M* point. When the SOC is included, three node-line rings become a pair of Dirac points. Our results for CaTe are consistent with their computations (Du *et al.*, 2017 ▸).

**Figure 8:**
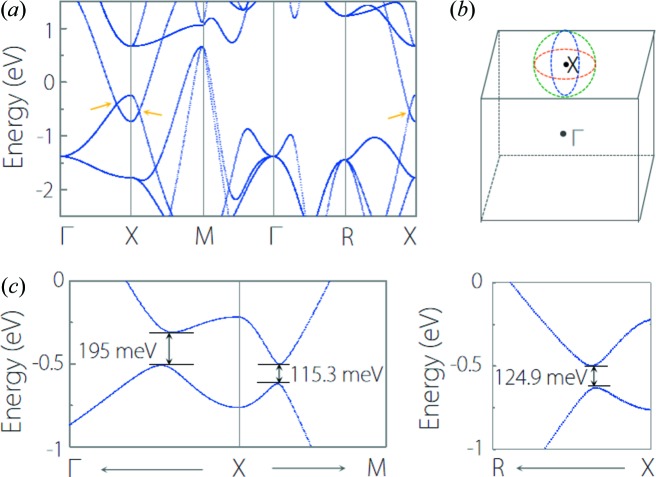
(*a*) Band structure of TiOs without SOC. (*b*) Schematic illustration of the three crossing nodal lines in TiO. (*c*) Enlarged band structure of TiOs with SOC at the Γ–*X*–*M* and *R*–*X* paths.

TiOs is another type-I nodal-line TM. As shown in Fig. 8 ▸(*a*), without the SOC effect, type-I band-crossing points occur at the Γ–*X*, *X*–*M* and *R*–*X* paths. Under the same protection mechanism with CaTe, these band-crossing points also belong to three crossing nodal lines. However, unlike CaTe, these nodal lines center on the *X* point instead of the *M* point, as shown in Fig. 8 ▸(*b*). With the SOC effect, these nodal lines are fully gapped to a sizeable gap (SOC-induced gap) along all the high-symmetry paths involving the *X* point, as shown in Fig. 8 ▸(*c*).

**Figure 9:**
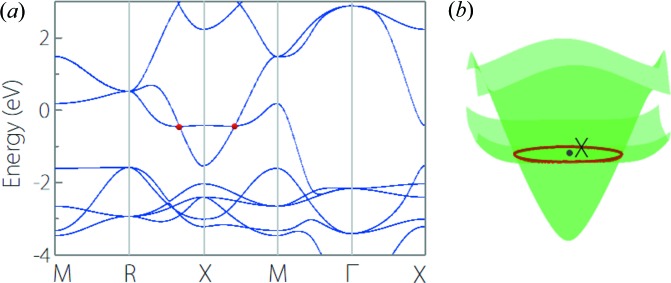
(*a*) Electronic band structure of CaPd. (*b*) Energy dispersion of the two crossing bands in (*a*). The red circle shows the critical-type nodal line in CaPd.

Aside from type-I nodal-line TMs, we have also identified critical-type nodal-line TMs in CsCl-type materials. Here, CaPd is discussed as a typical example and the band structure is shown in Fig. 9 ▸(*a*). It can be observed there are two band crossing points at the *R*–*X* and *X*–*M* paths. Different from CaTe and TiOs, the band-crossing points show a critical-type band-crossing feature, namely, one of the crossing bands is almost flat in CaPd. This band crossing produces a nodal line centering on the *X* point in the *k*_z_ = π plane. The three-dimensional energy dispersion of the two crossing bands is plotted in Fig. 9 ▸(*b*). We can observe from the critical-type nodal-line signature that the flat band is nearly non-dispersive near the nodal line at every *k*-path in the *k*_z_ = π plane. Such a critical-type nodal line was first proposed by Liu, Jin *et al.* (2018 ▸). Recently, they found that the crossing of a flat band and a dispersive band forms a critical-type nodal line which is protected by both mirror symmetry and the coexistence of *P* and *T* symmetries in CaPd. Our results are in good agreement with their report (Liu, Jin *et al.*, 2018 ▸).

**Figure 10:**
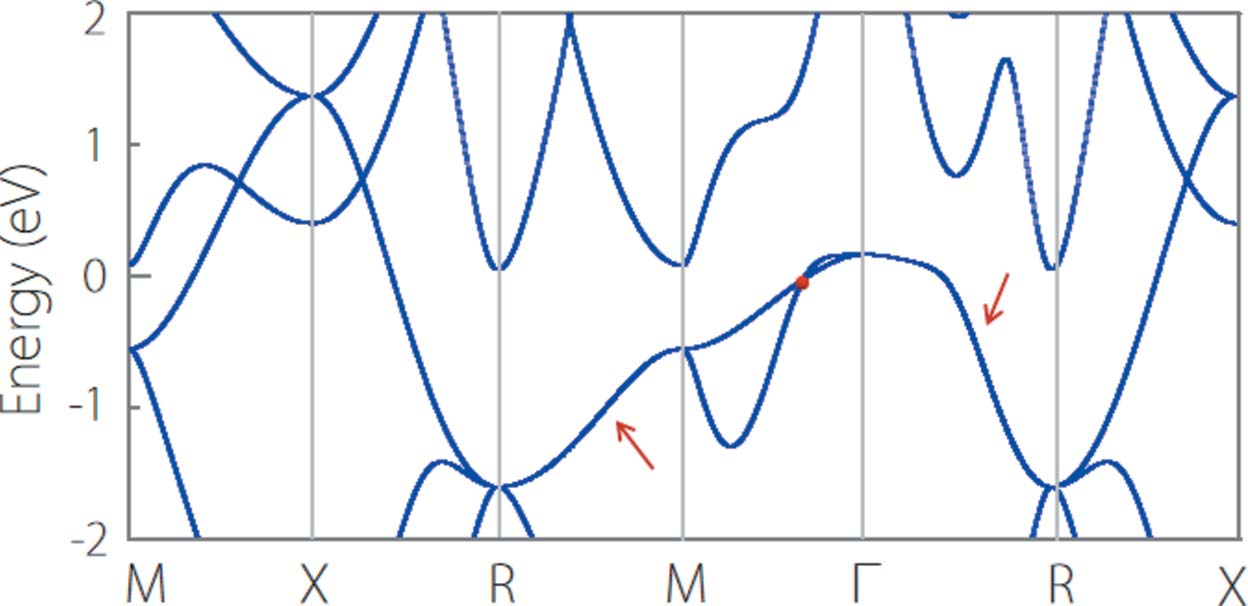
Electronic band structure of YCd. The red dot shows the type-II band crossing point at the *M*–Γ path. The red arrows point to the doubly degenerate bands along the whole *R*–*M* and Γ–*R* paths.

Some CsCl-type materials also show hybrid nodal-line band structures. Here we take YCd as an example; the band structure is shown in Fig. 10 ▸. We find that YCd has two types of band crossing: one type is the isolated type-II crossing point occurring at the *M*–Γ path; the other type is the doubly degenerate bands along the whole *R*–*M* and Γ–*R* paths, as indicated by the red arrows. The latter forms nodal lines which traverse across the whole BZ; we will not discuss this in detail here. In the following, we pay special attention to the band crossing point at the *M*–Γ path.

**Figure 11:**
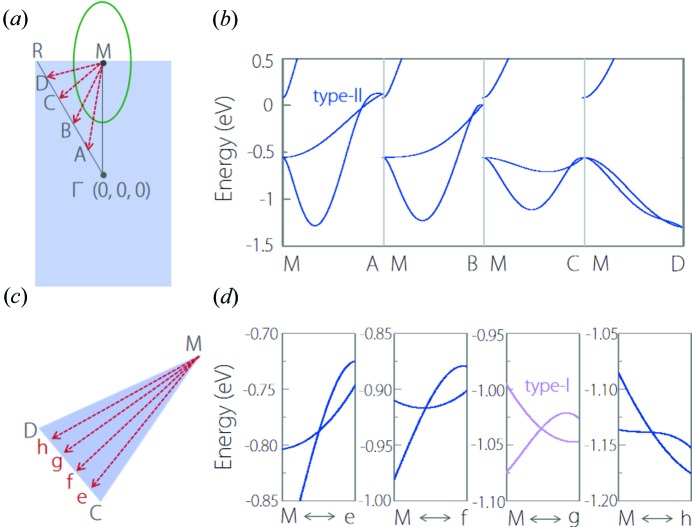
(*a*) Schematic illustration of the nodal line centering on the *M* point in the *R*–*M*–Γ plane. Crossing the nodal line, we choose four *k*-paths, namely *M*–*A*, *M*–*B*, *M*–*C* and *M*–*D*. The points *A*, *B*, *C* and *D* are equally spaced between Γ and *R*. (*b*) Electronic band structures of YCd at the *M*–*A*, *M*–*B*, *M*–*C* and *M*–*D* paths. (*c*) The selected *k* paths (*M*–*e*, *M*–*f*, *M*–*g* and *M*–*h*) between *M*–*C* and *M*–*D*. The points *e*, *f*, *g* and *h* are equally spaced between *C* and *D*. (*d*) Electronic band structures of YCd along the *M*–*e*, *M*–*f*, *M*–*g* and *M*–*h* paths.

**Figure 12:**
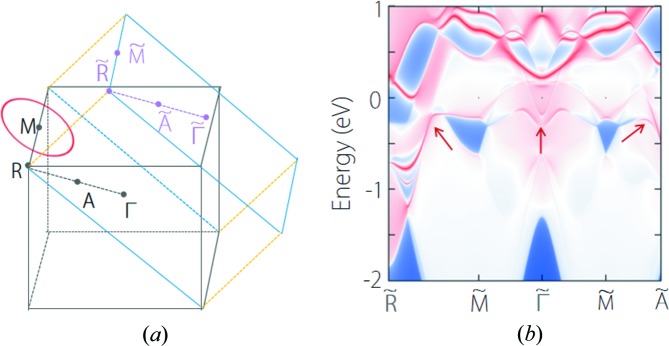
(*a*) Brillouin zone of the bulk and the (101) surface for YCd. The point *A* is the midpoint between Γ and *R*. The red circle denote the nodal line. (*b*) Projected spectrum on (101) surface. The red arrows point to the drumhead surface states.

We performed a careful investigation of the band structure around the type-II crossing point and found it belongs to a nodal line centering on the *M* point in the *R*–*M*–Γ plane, as shown in Fig. 11 ▸(*a*). For each *k*-path (*M*–*A*, *M*–*B*, *M*–*C* and *M*–*D*) from the *M* point in the plane, we can always obtain a type-II band crossing, as shown in Fig. 11 ▸(*b*). However, we noticed that the slopes of the two crossing bands between *M*–*C* and *M*–*D* paths have opposite sign. Thus we select another four *k*-paths [*M*–*e*, *M*–*f*, *M*–*g* and *M*–*h* as shown in Fig. 11 ▸(*c*)] between *M*–*C* and *M*–*D* to observe the evolution of the slope. The band structures along these paths are shown in Fig. 11 ▸(*d*). It is evident that, the band crossing along these *k*-paths exhibits a type-II–type-I–type-II transition. Therefore, the nodal line in Fig. 11 ▸(*a*) is a hybrid nodal line. In addition, we need to clarify that compared with common hybrid nodal lines, including Ca_2_As (Zhang, Yu, Lu *et al.*, 2018 ▸) and Li_2_BaSi (Zhang, Jin, Dai *et al.*, 2018 ▸), the type-I region is much smaller in YCd. So the nodal line in YCd can, to some degree, be viewed as a type-II nodal line. A nodal line is characterized by the drumhead-like surface states. For YCd, the BZ of a (101) surface and the surface spectrum are shown Figs. 12 ▸(*a*) and 12 ▸(*b*), respectively. We can observe clear surface bands on each *k*-path from the point.

Besides YCd, we found that CsCl-type ScCd and YMg also possess a hybrid nodal line near the Fermi level. It is worth noting that the realistic TMs with hybrid nodal lines are rarely reported [Ca_2_As (Zhang, Yu, Lu *et al.*, 2018 ▸) and Li_2_BaSi (Zhang, Jin, Dai *et al.*, 2018 ▸) are the only examples reported so far]. The proposed CsCl-type materials provide more choice on investigating the novel properties of hybrid nodal-line TMs.

### 3.4. TMs with the coexistence of a nodal line and triple nodal point   

**Figure 13:**
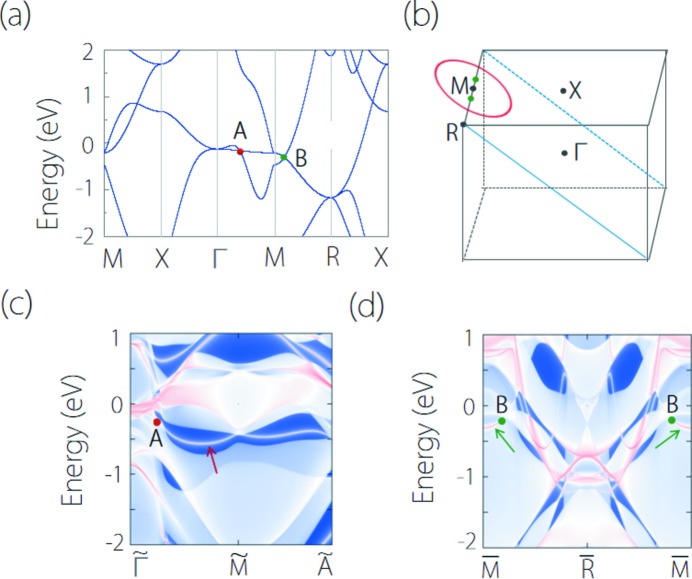
(*a*) Electronic band structure of YMg. The band crossing points at the Γ–*M* and *M*–*R* paths are denoting as point A and point B, respectively. (*b*) Brillouin zone of the bulk for YMg. The green points and red circle denote the position of the triple nodal point and nodal line. (*c*) (101) surface spectrum and (*d*) (001) surface spectrum for YMg. In (*c*) and (*d*), the surface bands originating from nodal line and triple nodal point are indicated by the arrows.

Several CsCl-type materials possess multiple types of band crossing. YMg is one of these materials The band structure of YMg is shown in Fig. 13 ▸(*a*). It manifests two band-crossing points near the Fermi level, which are denoted as point A and point B [see Fig. 13 ▸(*a*)]. The two nodal points have different degeneracy without counting spin: point A is doubly degenerate and point B is triply degenerate. By performing more detailed computations, we find point A resides on a hybrid nodal line centering on the *M* point, which is similar with that in YCd. Point B is a triple nodal point at the *R*–*M* path and is similar to that in YIr. As a result, a hybrid nodal line and a pair of triple nodal points coexist in YMg, as depicted in Fig. 13 ▸(*b*). Benefiting from the clean band structure near the nodal line and the triple nodal point, topological surface states for the nodal line and the triple nodal point can be clearly identified, as shown in Figs. 13 ▸(*c*) and 13 ▸(*d*), respectively.

## 4. Summary   

By using high-throughput computational screening based on first-principles, we have identified 61 TMs in existing CsCl-type materials. The identified TMs show rich topological character, including triple nodal point, type-I nodal line, critical-type nodal line and hybrid nodal line. In their band structures, the band crossing occurs near the Fermi level (|*E* − *E*_F_| < 0.5 eV). Most of the TMs identified show a clean topological band structure where there are no interfering bands around the band crossing. Moreover, these CsCl-type materials can be easily synthesized and are stable at room temperature, which greatly favors the experimental investigation of their topological properties.
